# Ewing Sarcoma of the Bone in Children under 6 Years of Age

**DOI:** 10.1371/journal.pone.0053223

**Published:** 2013-01-31

**Authors:** Maria Antonietta De Ioris, Arcangelo Prete, Raffaele Cozza, Marta Podda, Carla Manzitti, Andrea Pession, Elisabetta Schiavello, Benedetta Contoli, Rita Balter, Franca Fagioli, Gianni Bisogno, Loredana Amoroso, Franco Locatelli, Roberto Luksch

**Affiliations:** 1 Haematology-Oncology Department, Ospedale Pediatrico Bambino Gesù-IRCCS, Rome, Italy; 2 Paediatric Haematology-Oncology Department, University of Bologna, Bologna, Italy; 3 Paediatrics, Fondazione IRCCS Istituto Nazionale dei Tumori, Milan, Italy; 4 Paediatric Haematology-Oncology Department, Istituto Giannina Gaslini-IRCCS, Genoa, Italy; 5 Epidemiology Unit, Ospedale Pediatrico Bambino Gesù-IRCCS, Rome, Italy; 6 Paediatric Haematology-Oncology Department, University of Verona, Verona, Italy; 7 Paediatric Haematology-Oncology Department, Regina Margherita Hospital, Turin, Italy; 8 Paediatric Haematology-Oncology Department, University of Padua, Padua, Italy; 9 Pediatrics, University of Pavia, Pavia, Italy; Hospital Infantil Universitario Niño Jesús, Spain

## Abstract

**Background:**

Ewing Sarcoma Family Tumours (ESFT) are rare in early childhood. The aim of this study was to report the clinical characteristics and outcome of children under 6 years of age affected by ESFT of the bone in Italy.

**Methods:**

The records of all the children diagnosed with osseous ESFT in centres members of the Associazione Italiana di Ematologia ed Oncologia Pediatrica (AIEOP) from 1990 to 2008 were reviewed. The Kaplan–Meier method was used for estimating overall and progression-free survival (OS, PFS) curves; multivariate analyses were performed using Cox proportional hazards regression model.

**Results:**

This study includes 62 patients. An axial primary localization was present in 66% of patients, with the primary site in the chest wall in 34%. Fourteen (23%) patients presented metastatic disease. The 5-year OS and PFS were 73% (95% confidence interval, CI, 58–83%) and 72% (95% CI 57–83%) for patients with localized disease and 38% (95% CI 17–60%) and 21% (95% CI 5–45%) for patients with metastatic disease. Metastatic spread, skull/pelvis/spine primary localization, progression during treatment and no surgery predicted worse survival (P<0.01), while patients treated in the last decade had better survival (P  = 0.002). In fact, the 5-year OS and PFS for patients diagnosed in the period 2000–2008 were 89% (95% CI 71–96%) and 86% (95% CI 66–94%), respectively.

**Conclusion:**

The axial localization is the most common site of ESFT in pre-scholar children. Patients treated in the most recent period have an excellent outcome.

## Introduction

The Ewing Sarcoma Family Tumours (ESFT) are aggressive neoplasms due to proliferation of small round cells of neuroectodermal origin [1], [2]. ESFT are biologically characterized by the presence of a chimeric transcript, resulting from the fusion of the EWS gene with genes that encode for structurally related transcription factors, usually FLI1 or ERG 2 [3].

In 75% of cases, ESFT arise in bone and metastatic spread is present at diagnosis in 25% of patients. ESFT represent the second most common bone tumour in children and adolescents, accounting for 3% of all paediatric tumours [1], [2]. Significant progress has been achieved in the diagnosis and treatment of localised disease, over the past 30 years. Indeed, nowadays, overall survival (OS) is approximately 70% for patients with localized ESFT. However, OS still remains between 20% and 30% for patients with metastatic disease [1], [2], [4]–[6].

Poor outcome has also been reported to be associated with older age at presentation (age 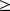
14 years or 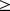
18 years) [4], [6], [7], larger tumour volume [6], [8], [9], poor response to induction therapy [6], axial tumour localization [4], [6], elevated serum levels of lactate dehydrogenase [10], less than 90% necrosis after primary chemotherapy [11], deletion of p16 [12] and mutation of p53 proteins [13], [14].

Both ESFT and osteosarcoma tumours have their highest incidence in late childhood/early adolescence, while occurrence in early childhood is rare [15]. The aim of this study is to describe the clinical characteristics and outcome of pre-scholar children affected by ESFT of bone diagnosed and treated in centres members of the Italian AIEOP (Associazione Italiana di Ematologia Oncologia Pediatrica).

## Methods

The AIEOP database, to which all cases of tumours diagnosed in member centres had to be routinely reported, was checked in order to identify patients aged between 0 and 18 years affected by ESFT of bone and diagnosed in the study period January 1990-March 2008.

Patient selection was made on the basis of a diagnosis of primitive bone tumour and a pathology compatible with ESFT; they represented a series of consecutive diagnoses during the study period. We excluded from analysis patients lacking a confirmatory histological diagnosis.

Sixty-two patients, diagnosed before 6 years of age, were identified and are included in the present analysis, the prevalence of pre-scholar ESFT in the AIEOP series was 14.6%.

For the study purposes, medical records of patients were retrospectively reviewed and data regarding gender, age, tumour localization at diagnosis, presence of metastases, site of metastases, tumour dimension, treatment protocol, degree of tumour necrosis histologically assessed after surgery and outcome were collected.

An informed written consent was obtained from patients’ parents or legal guardians at the time of diagnosis. All the therapeutic protocols were approved by local Institutional Review Board (IRB) and performed in accordance with the Helsinki declaration. This retrospective study was approved by the AIEOP board and by the Ospedale Pediatrico Bambino Gesù IRB.

### Statistical Methods

OS was defined as the time interval between the date of diagnosis and either the date of death from any cause or the date of last follow-up. Progression-free survival (PFS) was defined as the time interval between the date of diagnosis and the date of death, first relapse/progression or the date of last follow-up. The Kaplan–Meier method was used for the estimation of survival curves [16], while the log-rank test was used to compare differences between groups.

Multivariate analyses of variables potentially influencing OS and PFS were performed using Cox proportional hazards regression model. Variables that reached a P-value of 0.2 in univariate analysis were included in the initial model and variables were eliminated one at a time in a stepwise fashion, only keeping variables that reached a P-value of 0.05 or less into the final models. All P-values were 2-sided, with a type I error rate fixed at 0.05. Variables considered in risk factor analysis for OS and PFS were: the period of diagnosis (1990–1999; 2000–2008), gender, primary site (extremities, chest wall and other axial sites), presence of metastases, site of metastases (lung only, combined), tumour size (<8 cm, ≥8 cm), response to primary chemotherapy, surgery on primary tumour (yes/no), definitive surgery (yes/no); radiotherapy on primary tumour (yes/no); type of local control (none, radiotherapy alone, surgery alone, surgery plus radiotherapy) and necrosis after chemotherapy and surgery (100% or less than 100%). The cut-off for tumour size <8 cm/≥8 cm was chosen in view of the data published by Rodriguez Galindo et al. [4]. Definitive surgery was codified according to Krasin et al. [17]. Analyses were performed using the Stata 9.0 statistical software package (StatCorp LP, TX, USA).

## Results

### Patients and Treatment

Clinical characteristics of the 62 patients with bone ESFT and younger than 6 years are summarized in Table 1.

**Table 1:** Patient characteristics and univariate analysis of pre-treatment predictive factors.

		N %	PFS 5 yr	95% CI	P Value	OS 5 yr	95% CI	P Value
**Median Age**	42 months (5–70)		%			**%**		
**Gender**	Male	28 45	58	39–73	0,069	67	48–80	0,067
	Female	34 55	63	44–76		64	46–77	
**Stage of disease**	Localized	48 77	72	57–83	<0.0001	73	58–83	0.002
	Metastatic	14 23	21	5–45		38	17–60	
**Site of Metastasis**	Lung only	10 71	50	21–74	<0.02	58	27–80	0.140
	Combined	4 29	0			17	12–52	
**Tumor Size**	<8 cm	23 68	65	35–84	0.440	76	47–90	0.489
	≥8 cm	11 32	65	37–82		81	51–90	
**Primary Sites**	Extremity	20 32	68	43–84	0.043	73	47–88	0.002
	Chest Wall	21 34	73	46–88		89	63–97	
	Axial Sites (other thanchest wall)	21 34	39	17–61		45	23–65	

The median age at diagnosis was 42 months (range 5–70), 82% of patients being older than 24 months. The presenting symptoms were pain (32%), palpable lesion (36%), walking disorder or neurologic impairment (32%), respiratory symptoms (19%) and fever or/and anorexia (8%).

Forty-three patients were prospectively enrolled in the national protocols ongoing at the time of diagnosis; 10 patients were treated at the Fondazione IRCCS Istituto Nazionale dei Tumori (INT) in Milan, and 9 patients at the Ospedale Pediatrico Bambino Gesù (OPBG) in Rome with institutional protocols [18]–[21]. All protocols were based on cyclophosphamide, doxorubicin, ifosfamide, plus vincristine and/or actinomycin D. The main difference was the addition of etoposide in the more recent period. The INT protocol used cisplatin without doxorubicin, while the OPBG protocol used carboplatin.

Most patients (66%) had an axial primary tumour, with 34% having a chest wall primary localization; the prevalence of axial involvement was 75% and 57% in patients with either localized or metastatic disease, respectively (P = NS).There were no difference regarding the site distribution of primary tumours according to the period group: an axial primary tumour was diagnosed in 21/32 (67%) patients in the 1990–1999 period and in 21/30 (69%) patients in the later period 2000–2008.

Fourteen (23%) patients presented metastatic spread at diagnosis. According to primary site, metastatic disease was evident in 30% of patients with ESFT of the extremity, 14% of patients with ESFT of the chest wall and 25% of patients with ESFT of axial sites other than chest wall (P =  NS).

Nearly all patients (90%) received a local treatment at the site of primary tumour: it was surgery alone in 30 patients (48%); radiotherapy alone in 8 (13%) and surgery plus radiotherapy in 18 (29%).

Radical surgery was performed at diagnosis in 5 patients. Of the remaining 57, 56 were evaluable for response to primary chemotherapy and 6 of them progressed during chemotherapy. Out of these 6 patients who progressed during induction chemotherapy, 3 children underwent surgical removal of the primary tumour, while palliative radiotherapy was administered in one patient.

The radiotherapy dose was different according to primary site, ranging from 35 Gy to 60.4 Gy; 4 patients with lung metastases received whole lung irradiation at a dose of 12 Gy.

Twenty-four patients (39%) received myeloablative chemotherapy followed by autologous hematopoietic stem cell transplantation as consolidation therapy; 9 of them were affected by ESFT of the chest wall.

### Outcome and Analysis of Prognostic Factors

The median follow-up of the entire cohort was 62 months (range 1 month-25 years). Of the 62 patients, 20 (32%) died, 19 of relapsed/resistant disease and one patient of treatment-related complications after the second course of chemotherapy.

Secondary malignancies were not recorded.

Relapse/progression occurred in 25/62 (40%) patients after a median time from diagnosis of 19 months (range 2–121 months). Fourteen patients with localized disease (29%) experienced a relapse/progression, at a median time from diagnosis of 20 months (range 2–121 months), and 11 of them died. In the subgroup of patients with metastatic disease, 11/14 (79%) patients relapsed/progressed after a median time from diagnosis of 17 months (range 3–32 months) and 8 of them died due to disease progression.

Tables 1 and 2 summarize the results of univariate analysis of factors influencing patients’ outcome. Metastatic spread at time of diagnosis, combined metastasis and a primary tumour localized in the skull, pelvis or spine were found to be associated with worse OS and/or PFS.

**Table 2:** Univariate analysis of treatment predictive factors.

		n	PFS 5 yr	95% CI	Univariate analysis	OS 5 yr	95% CI	Univariate analysis
			%		*p* value	%		*p* value
**Treatment Period**	1990–1999	32	38	21–55	<0.001	48	30–64	0.002
	2000–2008	30	86	66–94		89	71–96	
**Histological Response**	100% necrosis	15	87	56–96	0.3562	100		0.058
	<100%	15	68	36–87		72	34–90	
**Surgery**	No	14	17	3–42	<0.001	21	5–45	<0. 001
	Yes	46	74	58–85		83	68–92	
**Definitive Surgery**	No	25	42	22–61	0.002	42	22–60	<0. 001
	Yes	35	75	56–87		87	69–95	
**Response to CT**	PD	6	0		<0.0001	0		<0. 001
	No PD	49	68	52–79		75	59–85	
**Local Control**	None	4	0		<0.001	25	1–66	<0. 001
	RT alone	8	29	4–61		25	4–56	
	Surgery alone	30	72	52–85		82	62–92	
	Surgery plus RT	18	69	40–86		75	46–90	

Legend: PD, progression of the disease; CT, Chemotherapy; RT, radiotherapy.

**Figure 1:**
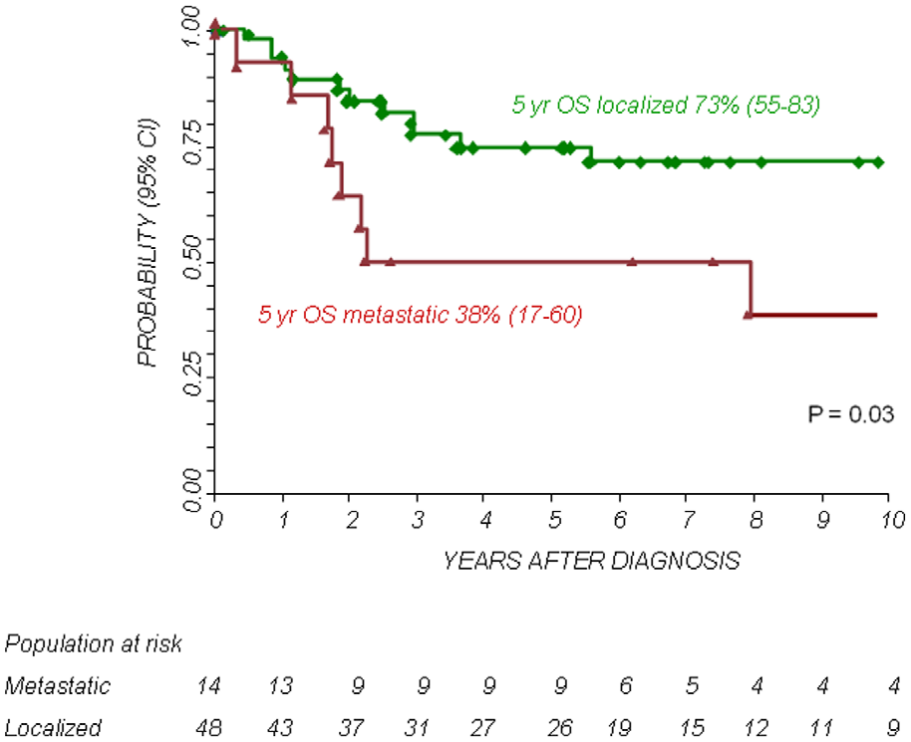
Overall Survival (OS) according to stage (localized or metastatic disease).

**Figure 2:**
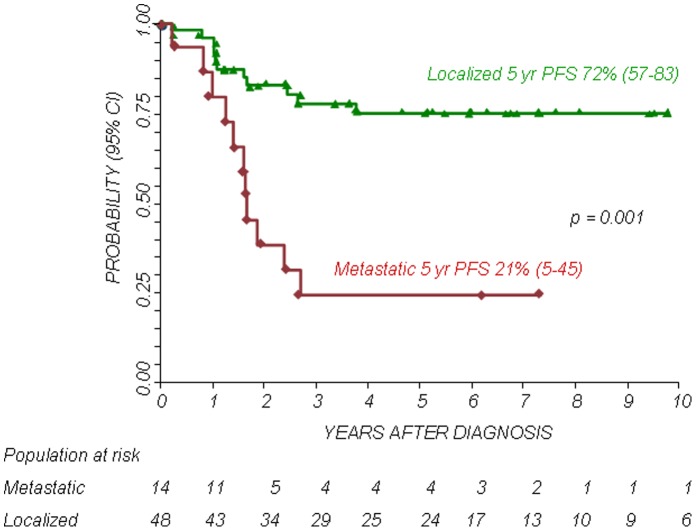
Progression Free Survival (PFS) according to stage (localized or metastatic disease).

**Figure 3:**
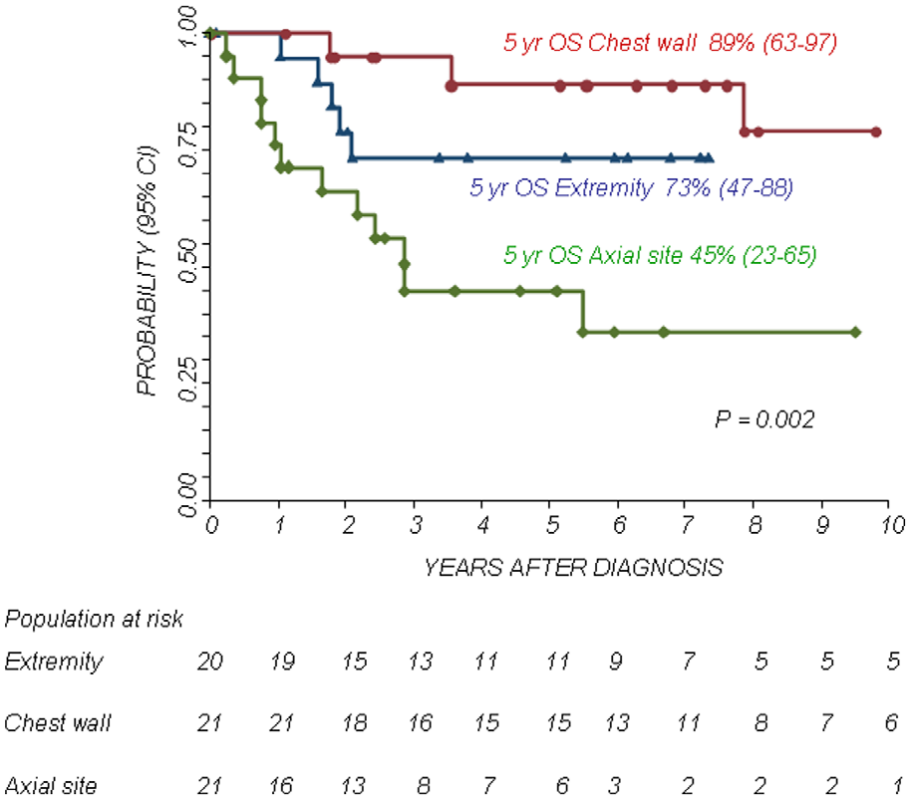
Overall Survival (OS) according to primary site.

**Figure 4:**
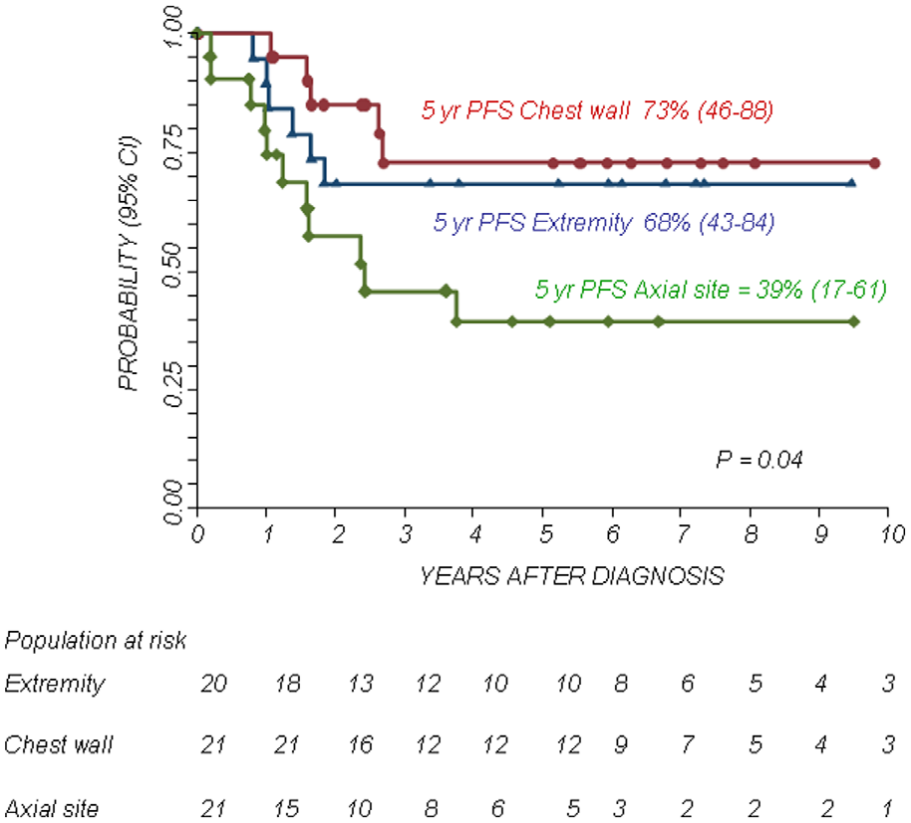
Progression Free Survival (PFS) according to primary site.

The 5-year Kaplan-Meier estimates of OS and PFS for patients with localized disease were 73% (95% confidence interval CI 58–83%) and 72% (95% CI 57–83%), respectively. The 5-year Kaplan-Meier estimates of OS and PFS for patients with metastatic disease was 38% (95% CI 17–60%) and 21% (95% CI 5–45%), respectively. The difference in OS and PFS between the two patient groups was statistically significant (P<0.01). The 5-year OS and PFS for patients with primary chest localization were 89% (95% CI 63–97%) and 73% (95% CI 46–88%), respectively, while the 5-year OS and PFS for other axial sites were 45% (95% CI 23–65%) and 39% (95% CI 17–61%), respectively and those for patients with a primary localization at the extremities were 73% (95% CI 47–88%) and 68% (95% CI 43–84%), respectively. The differences in OS and PFS between the groups were statistically significant (P<0.05. see also figure 1, 2, 3, 4).

The OS of all AIEOP patients with ESFT, irrespectively of age, was 60% (95% CI 57–63) for patients with localized disease, 32% (95% CI 28–36) for patients with metastatic disease and 53% (95% CI 46–59) for patients with primary chest tumour. In our cohort of children below the age of 6, patients affected by primary chest wall involvement presented an excellent outcome; this favourable outcome is probably due to a limited proportion of patients with metastatic spread and an aggressive strategy for local control. Indeed, surgery plus radiotherapy was employed in 38% of patients and more than 40% of children received high dose chemotherapy (see Table 3 and 4 for details).

**Table 3:** Characteristics of patients with chest wall primary tumour.

Pt	Period of diagnosis	Sex	Age at diagnosis (in months)	Metastasis	Tumor Size (in cm)	Necrosis 100%	Local control	Definitive Surgery	Treatment	Survival	Relapse
1	2000–2008	F	59	no	<8	na	Surgery plus RT	no	CT-Surgery-RT	Alive, 66 mo	no
2	2000–2008	M	12	no	>8	yes	Surgery	yes	CT-Surgery-HDC	Alive, 82 months	no
3	2000–2008	F	29	no	<8	no	Surgery plus RT	yes	CT- Surgery-RT-HDC	Alive, 87 months	no
4	1990–1999	F	48	no		yes	Surgery	yes	CT-Surgery	Alive, 132 months	yes, 20 months
5	1990–1999	F	45	no	>8	no	Surgery	yes	CT-Surgery	Died, 42 months	yes, 32 months
6	2000–2008	M	35	no	>8	no	Surgery plus RT	yes	CT- Surgery-RT-HDC	Alive, 29 months	no
7	2000–2008	M	55	no	<8	yes	Surgery	yes	CT-Surgery	Alive, 91 months	no
8	1990–1999	F	13	no	<8	na	Surgery	no	CT-Surgery	Alive, 214 months	yes, 121 months
9	2000–2008	F	38	no		yes	Surgery	yes	CT-Surgery-HDC	Alive, 29 months	no
10	2000–2008	F	65	no	<8	yes	Surgery plus RT	yes	CT- Surgery-RT-HDC	Alive, 75 months	no
11	1990–1999	F	11	Yes, lung	>8	yes	Surgery	yes	CT-Surgery-HDC	Alive, 128 months	no
12	1990–1999	F	67	no		no	Surgery	yes	CT-Surgery	Alive, 62 months	no
13	2000–2008	M	41	no	>8	no	Surgery plus RT	yes	CT- Surgery-RT-HDC	Alive, 22 months	no
1	1990–1999	F	28	no	>8	yes	Surgery	yes	CT-Surgery	Alive, 165 months	yes, 71 months
15	1990–1999	M	55	no	<8	no	Surgery	yes	CT-Surgery	Alive, 117 months	no
16	1990–1999	F	18	Yes, lung		na	None		CT-HDC	Died, 94 months	yes, 19 months
17	2000–2008	F	36	no	>8	no	Surgery	yes	CT-Surgery	Alive, 13 months	no
18	2000–2008	F	65	no	>8	yes	Surgery plus RT	yes	CT-Surgery-RT	Alive, 97 months	no
19	1990–1999	M	43	Yes, lung	<8	na	Surgery plus RT	yes	CT- Surgery-RT-HDC	Alive, 147 months	yes, 32 months
20	1990–1999	M	36	no		na	Surgery plus RT	no	CT-Surgery-RT	Died, 21 months	yes, 13 months
21	1990–1999	F	42	no	<8	no	Surgery	yes	CT-Surgery-HDC	Alive, 126 months	no

Legends: na, not available; M, male; F, female; RT, radiotherapy; CT, chemotherapy; HDC, High Dose Chemotherapy.

**Table 4:** Type of local control according to primary site of the tumour.

	Pt	None	Surgery Alone	RT alone	Surgery plus RT
**Extremity**	20	5,0%	70,0%	5,0%	20,0%
**Chest Wall**	21	4,7%	57,0%	0,0%	38,0%
**Axial Sites (other than chest wall)**	21	4,7%	29,0%	33,0%	28,6%

**Figure 5:**
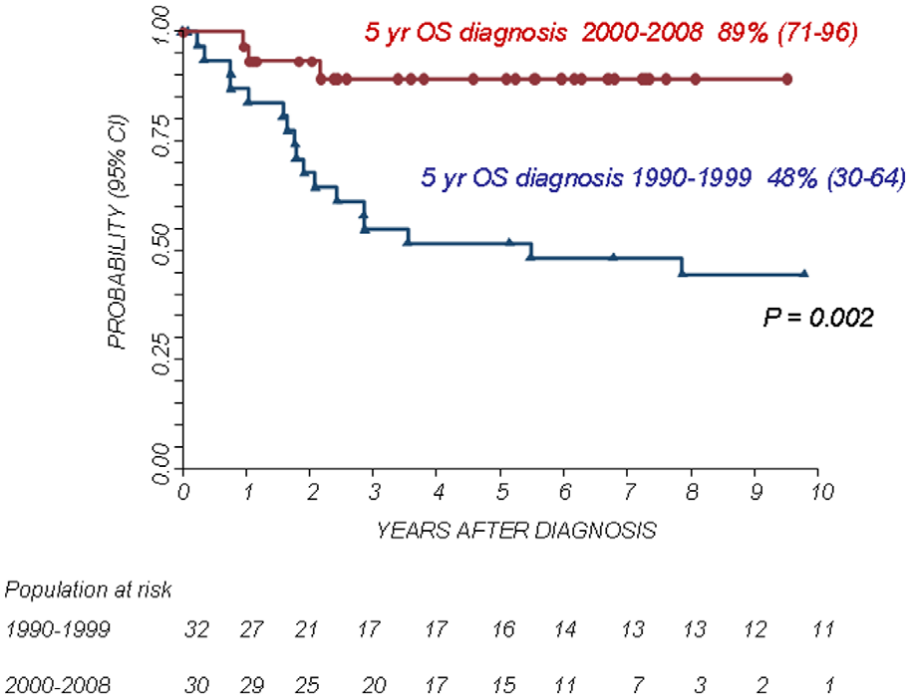
Overall Survival (OS) according to the period of diagnosis.

**Figure 6:**
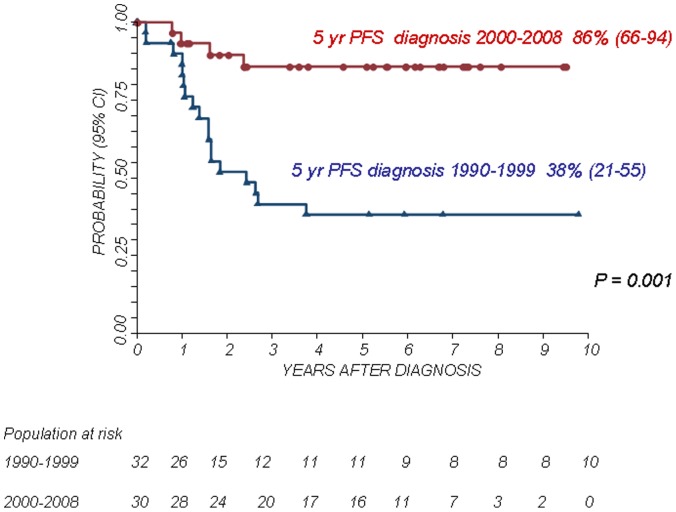
Progression Free Survival (PFS) according to the period of diagnosis.

The treatment period (1990–1999 versus 2000–2008) influenced outcome (see Figures 5 and 6). In fact, although there were no statistically significant differences between the two groups in terms of metastatic spread (28% in the 1990–1999 period versus 17% in the later period, P = 0.3), the outcome of children treated in the more recent period was better. In detail, the 5-year OS and PFS for patients diagnosed in the period 2000–2008 was 89% (95% CI 71–96%) and 86% (95% CI 66–94%), respectively, while in the previous period the 5-year OS and PFS were 48% (95% CI 30–64%) and 38% (95% CI 21–55%), respectively (P<0.01 for both OS and PFS). The treatment attitude throughout the two periods analyzed was different. Indeed, in the first period surgery was performed in 67% of patients while in the more recent period, this percentage rose up to 94% (P = 0.002). Also the general strategy for disease control was different with a clear evidence of a more aggressive and a multi-modality approach based on conventional chemotherapy, combined surgery and radiotherapy (Table 5). As expected, the surgical and local control approach was different according to primary site; surgery alone was used in 70% of extremity primary and this percentage falling down to 29% in patients affected by primary axial site (other than chest wall) (see Table 4 for details about local control strategy by primary site).

**Table 5:** Treatment strategy by Treatment Period.

Treatment Period	1990–1999	2000–2008
	%	%
**CT**	10	3
**CT plus RT**	23	3
**CT plus Surgery**	50	50
**CT plus Surgery and RT**	17	44

Legend: CT, chemotherapy, RT, radiotherapy.

In univariate analysis, surgery represented a major favourable prognostic factor for both survival and recurrence (P<0.01). Patients who received treatment with surgery or surgery plus radiotherapy were found to have better outcomes than those who were treated with radiotherapy alone (P<0.01). As expected, progressive disease during first-line treatment represented a major adverse prognostic factor (Table 6).

**Table 6:** Multivariate analysis of pre- and post-treatment predictive factors.

		HR PFS	95 % CI	Munivariate analysis	HR OS	95 % CI	Multivariate analysis
				p value			p value
**Stage of disease**	Metastatic vs Localized	5.3	2.2–2.8	0.00	2.9	1.1–7.8	0.03
**Primary Sites**	Axial Sites vs Other sites	4.0	1,3–2,6	0.02	12	2.5–7.8	0.002
**Response to CT**	PD vs no PD				19.9	2.4–64	0.05

Only the variables with a statistically significant p value are shown.

Legend: PD, progressive disease; CT, chemotherapy.

In the final model of multivariate analysis, the presence of metastasis and a primary tumour of spine, skull or pelvis were poor prognostic factors for both OS and PFS. Of the several treatment-related variables found to predict outcome in univariate analysis, only progression during first-line chemotherapy remained significant in multivariate analysis (Table 6).

## Discussion

Bone tumours are rare in pre-scholar children. In a series of 1474 paediatric bone tumours, patients under the age of 6 accounted for only 5,8% [22].

Ewing Sarcoma represents the second most frequent bone tumour after osteosarcoma: the highest incidence was observed in late childhood and adolescence [15]. Two recent papers presented clinical data and outcome of pre-scholar children affected by osteosarcoma: in both papers, the authors reported a peculiar histological pattern and an higher incidence of mutilating surgery in younger patients, while the outcome was not statistically different from that of older children [23]–[24].

The incidence of ESFT in early childhood is rare, accounting for less than 10 cases per million each year, while the incidence of this neoplasm is about 30–40 cases per million between 11 and 18 years of age [15]. In a recent paper, the ESFT rate in different paediatric age groups was presented: no case was reported in the first year of age while the incidence rate between 1 and 6 years ranged from 0.99 to 2.04 per million of children (22). In view of this observation, it is not surprising that data about the clinical characteristics and outcome of ESFT in early childhood were limited.

The aim of this study was to report on the clinical characteristics and outcome of pre-scholar children affected by bone ESFT in Italy. The main study limitation is the retrospective analysis carried out on a population diagnosed over a long period of time and treated according to different protocols in several paediatric centres. Moreover, the tumour size and data on tumour dimensions were available only in about 70% of patients (44/62) and had been recorded with different imaging tools (either CT or MRI). The criterion of 100% necrosis vs. less than 100% was chosen in order to limit the differences in the evaluation within the AIEOP pathology panel. Nevertheless, considering the low occurrence rate of bone ESFT in pre-scholar children, we believe that the present study provides useful information on a rare subgroup of patients.

In the AIEOP experience, the prevalence of bone ESFT diagnosed before the age of 6 years was 14.6% of all paediatric ESFT patients (i.e. aged less than 18 years); this finding may be influenced by the fact that we collected data only from paediatric centres and we cannot exclude that some of the older patients with ESFT have been referred to Institutions not reporting to AIEOP.

In this cohort, most patients had an axial primary localization (66%, for the whole group, being 75% and 57% in patients with either localized or metastatic disease, respectively), while, in other series the axial site represents only 50% [7]–[9], [18], [24]–[26] of cases. As observed in infants [27], the axial site seems to be a peculiar characteristic of younger age. Recently, Van den Berg reported a series of 14 infants: all patients had an axial tumours and most of them had peripheral neuroectodermal tumours (PNETs) [27].

Also chest wall tumours seem to be characteristic of younger age: we found a prevalence of 34%, while a prevalence of less than 20% has been reported [7]–[9]; [18], [25]–[26]. Pelvis tumours occurred in only 11% of patients and, as observed in a large series, the incidence of pelvis tumours increases with age [26].

The outcome of our cohort, both in terms of OS and PFS. is comparable with that observed in older patients. In literature, age at diagnosis emerges as a significant prognostic factor for ESFT [4], [6], [7], [26] as also recently observed in multifocal disseminated ESFT patients enrolled in EUROEWING 99 Protocol [28] and age was considered in the risk stratification proposed by Rodriguez Galindo [4].

Survival showed an impressive improvement in the last decade, with OS exceeding 85% in comparison with a value of less than 50% of the previous decade (P<0.002), considering both localized and metastatic patients. The two groups presented comparable clinical features: in particular, there were no differences in term of metastatic spread and/or primary tumour site, while a difference in strategy was clearly evident between the two time periods. The favourable outcome achieved in the last decade is possibly due to a multi-disciplinary and more aggressive strategy based on surgery and radiotherapy. Moreover, a more aggressive surgical approach is observed in the recent period: in the group 2000–2008, 94% of patients underwent surgical removal of primary tumour, while surgery was performed in 67% of patients in the group 1990–1999.

In selected case conventional chemotherapy followed by high-dose treatment and stem cell support as consolidation treatment was used, while exclusive radiotherapy as local treatment was deserved only to very few cases. Pelvic localization, poor histological response, metastases, surgery, quality of local control of disease and response to treatment have been reported to influence the outcome [4], [6]–[9], [11], [25], [26].

In our cohort, the site of primary tumour and the presence of metastases resulted to have an impact on survival in the multivariate analysis. In contrast to the results reported by Van den Berg et al. [27], our data confirm the role of the previously reported prognostic factors [4], [6]–[11]. Furthermore, our data indicate that a favourable outcome is presently attainable both in younger patients affected by non-metastatic ESFT and in chest ESFT with an aggressive treatment strategy. In the present series, the chest wall primary represents a third of the population with an excellent outcome, probably due to an aggressive strategy based on surgery plus radiotherapy for local control and high-dose chemotherapy.

A further prospective analysis on a larger number of patients with localized or metastatic ESFT, homogeneously enrolled in the most recent protocols, should answer the question whether age does or does not have a prognostic value, independently from its association with other variables predicting a poor outcome. Moreover, further studies are warranted to provide information on the biological aspects and to possibly explain the different pattern of primary tumour localization.

### Conclusion

ESFT is confirmed to be a rare tumour in early childhood. In the AIEOP experience about 15% of affected children are younger than 6 years of age while the axial -in particular chest- localization is the most common primary site. In this group, the role of previously reported prognostic factors was confirmed and a favourable outcome is attainable with an aggressive strategy.
